# Light/heat effects on RNA editing in chloroplast NADH-plastoquinone oxidoreductase subunit 2 (*ndhB*) gene of *Calotropis* (*Calotropis procera*)

**DOI:** 10.1186/s43141-020-00064-4

**Published:** 2020-09-11

**Authors:** Ahmed M. Ramadan

**Affiliations:** 1 Department of Biological Sciences, Faculty of Science, King Abdulaziz University (KAU), PO Box 80141, Jeddah, 21589 Saudi Arabia; 2 Department of Plant Molecular Biology, Agricultural Genetic Engineering Research Institute (AGERI), Agriculture Research Center (ARC), Giza, Egypt

**Keywords:** Light, RNA editing, *ndhB*, Desert plant, *Calotropis* (*Calotropis procera*)

## Abstract

**Background:**

RNA editing is common in terrestrial plants, especially in mitochondria and chloroplast. In the photosynthesis process, NAD dehydrogenase plays a very important role. Subunit 2 of NADH-dehydrogenase is one of the major subunits in NAD dehydrogenase complex. Using desert plant *Calotropis* (*Calotropis procera*), this study focuses on the RNA editing activity of ndhB based on light time.

**Results:**

*NdhB* (NADH-dehydrogenase subunit 2) gene accession no. MK144329 was isolated from *Calotropis procera* genomic data (PRJNA292713). Additionally, using RNA-seq data, the cDNA of the *ndhB* gene of *C. procera* was isolated at three daylight periods, i.e., dawn (accession no. MK165161), at midday (accession no. MK165160), and pre-dusk (accession no. MK165159). Seven RNA editing sites have been found in several different positions (nucleotide no. C467, C586, C611, C737, C746, C830, and C1481) within the *ndhB* coding region. The rate of these alterations was deferentially edited across the three daylight periods. RNA editing rate of *ndhB* gene was highest at dawn, (87.5, 79.6, 78.5, 76, 68.6, 39.3, and 96.9%, respectively), less in midday (74.8, 54.1, 62.6, 47.4, 45.5, 47.4, and 93.4%, respectively), and less at pre-dusk (67, 52.6, 56.9, 40.1, 40.7, 33.2, and 90%, respectively), also all these sites were validated by qRT-PCR.

**Conclusion:**

The differential editing of chloroplast *ndhB* gene across light periods may be led to a somehow relations between the RNA editing and control of photosynthesis.

## Background

RNA editing is one form of post-transcriptional changes, which include nucleotide insertion, deletion, or modification [1, 2]. This mechanism was first discovered in mitochondrial trypanosomes [3]. The typical form of animal RNA editing is the alteration of adenosine (A) to inosine (I) which is interpreted as guanosine, this is achieved through the activity of adenosine deaminases [1]. Also, the conversion of cytidine to uridine was reported in the apolipoprotein B gene and produced by (apobec/ACF) editosome [4]. Terrestrial plants RNA editing is common in mitochondrial and chloroplast genes. Cytidine to uridine nucleotide conversion is observed in most transcripts plant organelle [5, 6]. Forty-three editing sites in the chloroplast genome [7] and 619 sites mitochondrial genome [8] were investigated in *Arabidopsis* (*Arabidopsis thaliana*). RNA editing is found in coding sequences, long non-coding RNAs, introns, tRNAs, microRNAs, and rRNAs [9, 10]. Most RNA editing cases in coding regions where integrated biological functions occur in the first or second codon position [11]. The efficiency of chloroplast transcript editing varies among developmental stages, tissue types, environmental factors, and ecotypes [12, 13]. Many reports have investigated RNA editing in terrestrial plant mitochondria genes, such as cytb, *nadI*, *nad3*, *coxII*, and *coxIII* [14, 15]. In ferns, reverse alterations T to C were identified in *atp1*, *nad5*, *rps1*, and *rpl2* genes [16].

In chloroplast, the activity of NADH dehydrogenase (NDH) complex increases under various environmental conditions, e.g., drought, temperature, and high light stress [17]. RNA editing sites of the ryegrass plastome were investigated in genes encoding NDH proteins, in particular the *ndhB* gene [18].

In recent years, next-generation sequencing (NGS) has a rapid and economical way to find organelle RNA editing sites, and extensively examine the whole transcriptome [8, 10]. Besides these advantages in wide RNA editing detection, NGS technology avoids the limitations of the Sanger method to accurately detect RNA editing in tissue or low-level editing sites that have not been detected before. For example, NGS as well as bioinformatics tools have allowed researchers to identify many novel chloroplast editing sites in *Arabidopsis* [7, 8, 19].

In our study, we aim to explore the relationship between the light/heat conditions and RNA editing gene wild plants. So, we used RNA-seq data of *Calotropis procera* leaf tissues to explore these effects.

## Methods

### Plant material and RNA extraction

Triplicates plants of *C. procera* were used in this study. Three biological leaf disks from each plant per time are mixed*.* Plants were collected from the KSA western region (Saudi Arabia, latitude 22° 16′ 49.55, longitude 39° 7′ 28.18). Three samples were isolated at three different times (dawn, midday, and pre-dusk). Light density/heat temperature was 10 lux/35 °C at dawn, 9000 lux/45 °C at midday, and about 95 lux/37 °C at pre-dusk. Total RNA was isolated according to Ramadan and Hassanein [20]. RNA concentration was measured at OD 260 nm. Also, the DNA free RNA quality was evaluated by RT-PCR and PCR before sequencing. Samples were deep sequenced in (BGI) Beijing Genomics Institute, China.

### Next-generation sequencing (NGS)

Paired-end short-sequence reads from *C. procera* were developed with Illumina Genome AnalyserIIX (GAIIx). A minimal of 50-bp read with a confidence interval of 95% was considered for the study. Libraries from leaf tissues were constructed, generating the following raw sequencing reads number 158,537,240 at dawn, 108,554,060 at midday, and 108,751,224 at pre-dusk with average number of reads that align to known reference bases of 128.72 bp, 128.69 bp, and 119.37 bp, respectively.

The RNA sequence reads were submitted to NCBI BioProject with accession numbers PRJNA531450 for dawn transcriptome, PRJNA531451 for midday transcriptome, and PRJNA531452 for pre-dusk transcriptome. RNA editing site was detected by CLC Genomic Workbench 3.6.5 [21]. Mapping parameters were adjusted at a similarity and length fraction of 98 percent. The reads data were mapped to the plastome of *Calotropis procera* (accession no. MG678914) [22]. The low-frequency variant 5% was optimized for a significant. The minimum coverage was 20, the minimum count was 4, and the minimum frequency was 5. In addition, the coverage depth of RNA editing sites and total read counts were reported. The nucleotide conversion frequency of each site was assessed by the number of reads for the conversion of nucleotides divided by total reads [23].

#### RNA editing and amino acids analysis

All obtained *ndhB* sequences, genomic as well as cDNA sequences, were analyzed for RNA editing and corresponding protein using multi-sequence alignment through CLC genomic workbench 3.6.5.

### Domain analysis

NCBI’s conserved domain database (CDD) was used to identify the functional domains by submitting the coding sequence of obtained *ndhB* gene sequence (http://www.ncbi.nlm.nih.gov/Structure/cdd/wrpsb.cgi).

### RNA editing site validation and analysis using RT-qPCR

To validate predicted editing sites, biological triplicates of leaf tissue samples were used. Total RNA was extracted from all the samples using Qiazol (Qiagen, Cat No. 79306).

Each 20 μl cDNA synthesis reaction was containing 1 μg of total RNA, 1 μM poly dT oligonucleotide (Biolegio, Nijmegen, Netherlands), 10 μl M-MuLV reaction Mix, 2 μl M-MuLV enzyme (New England Biolabs), and then incubation at 42 °C for 45 min. The RT-PCR reaction was enforced according to Mohammed et al. and Rodrigues et al. [24, 25]. qRT-PCR has a volume of 20 μl reaction containing 10 μl SsoAdvancedTM Universal® SYBR Green Supermix (Bio-RAD, USA), 1 μl cDNA, and 10 μM each forward and reverse primers (Table 1). *Calotropis procera* actin gene was used as a reference to normalize data. Applied Biosystems Real-Time PCR (Thermo Fisher Scientific, USA) are used to carry out the following conditions: 5 min at 94 °C, 40 cycles of 20 s at 95 °C, and 1 min at 56 °C. Dissociation curve analysis was carried out by heating at 95 °C for 60 s; 55 °C for 30 s, and 0.2 °C increase per cycle till 95 °C. PCR amplification was done using primers designed by Primer-Blast (https://www.ncbi.nlm.nih.gov/tools/primer-blast/). Two forward specific primers with different 3′ ends (original and substitute nucleotide) and one reverse primer were designed for each editing site (Table 1). The percentage of RNA editing was performed according to [25]:

$$ \% RNA\kern0.5em editing=\frac{2\left(\mathrm{Ct}\ \mathrm{mean}\ \mathrm{of}\ \mathrm{T}\ \mathrm{variant}-\mathrm{Ct}\ \mathrm{mean}\ \mathrm{of}\ \mathrm{C}\ \mathrm{variant}\right)}{\left\{2\left(\mathrm{Ct}\ \mathrm{mean}\ \mathrm{of}\ \mathrm{T}\ \mathrm{variant}-\mathrm{Ct}\ \mathrm{mean}\ \mathrm{of}\ \mathrm{C}\ \mathrm{variant}\right)+1\right\}}\times 100 $$

**Table 1:** Description of forward (5′-3′) and reverse (3′-5′) primer sets were designed as two forward specific primers with different 3′ ends (original and underlined substitute nucleotide) and one reverse primer site except for position 1481, two specific reverse and one forward

Position	F 5′ → 3′	R 3′ → 5′
467	TAACTATCTTTGTAGCTC**T**	ATGAAATATTTACTCATGGG
	TAACTATCTTTGTAGCTCC	
586	AAGCTCTTCTATTCTGGTT**T**	TCTCTCCCCCGGATAAACCAT
	AAGCTCTTCTATTCTGGTTC	
611	TTCTCTTGGCTATATGGTT**T**	CTGGAGTGGGAGATCCTTCG
	TTCTCTTGGCTATATGGTTC	
737	ATTGGGTTCAAGCTTTCCC**T**	TGGAGTGGGAGATCCTTCGT
	ATTGGGTTCAAGCTTTCCCC	
747	AGCTTTCCCCAGCCCCTTC**T**	GGCATCTTCTTCTGGAAATC
	AGCTTTCCCCAGCCCCTTCC	
830	ACTTCGAAAGTAGCTGCTT**T**	AACGTATGCTTGCATATTCG
	ACTTCGAAAGTAGCTGCTTC	
1481	CGAAACCAAGAAATAACCCCT	CGGGTTCATTGATATTCCT**A**
		CGGGTTCATTGATATTCCT**G**
*Actin*	TGGTCGTCCAAGACACACTG	CTCTTCAGGGGCAACACGAA

ANOVA test was conducted in SPSS (20.0; IBM) to assess the variation of editing levels per time. *P* values < 0.01 were considered significant according to Tukey’s test.

### 3D structure alignment

Modeling pairwise alignment was achieved by I-TASSER, (Iterative Threading ASSEmbly Refinement) for three ndhB proteins (dawn, midday, and pre-dusk) as well as correspondence protein of DNA coding sequence. This approach predicts protein structure and function [26].

## Results

### Identification of *C. procera ndhB* gene

The *Calotropis procera ndhB* gene was characterized via this study (accession number MK144329) using short DNA sequence reads 71,349,934 paired ends (BioSample Accession Numbers PRJNA292713). *Calotropis procera* chloroplast gene *ndhB* (accession no. MG678914) of *t* was used as reference in CLC genomic workbench. Multi-sequence alignment was enforced using the best hits of BLAST search (Table 2, Fig. S1).

**Table 2:** Accession number for each DNA sequence, description, organism name, T.S., % ident., and *E* value of homologous sequence to *C. procera ndhB* gene sequence identified using BLAST programs

Accession number	Description	T.S.	% ident.	*E* value
MH939982	*Calotropis procera* chloroplast	8190	100	0.0
MH939981.1	*Calotropis gigantea* chloroplast	8190	100	0.0
KF539850.1	*Matelea biflora* plastid, partial genome	4095	100	0.0
MG678915.1	*Pergularia daemia* voucher OKLA chloroplast	4089	99	0.0
MG678876.1	*Asclepias mellodora* var. mellodora	4089	99	0.0
MG678856.1	*Asclepias pilgeriana* chloroplast, partial genome	4089	99	0.0
MG678843.1	*Asclepias boliviensis* chloroplast, partial genome	4089	99	0.0
MG678835.1	*Asclepias aff. aequicornu* chloroplast, partial genome	4089	99	0.0
KF539853.1	*Telosma cordata* plastid, partial genome	4089	99	0.0
KF539852.1	*Sisyranthus trichostomus* plastid, partial genome	4089	99	0.0

*T.S.* total score, *% ident.* identity percentage

### Characterization of *ndhB* mRNA

cDNA *ndhB* gene of *C. procera* at dawn (accession no. MK165161), at midday (accession no. MK165160), and at pre-dusk (accession no. MK165159) were characterized using RNA-seq raw data. A total of 215,841,902 paired-end short sequence reads of the RNA *C. procera* were generated. In a CLC genomic workbench, *ndhB* gene (accession no. MK144329) of *C. procera* was used as a template**.**

### RNA editing in *ndhB* transcript

**Fig. 1:**
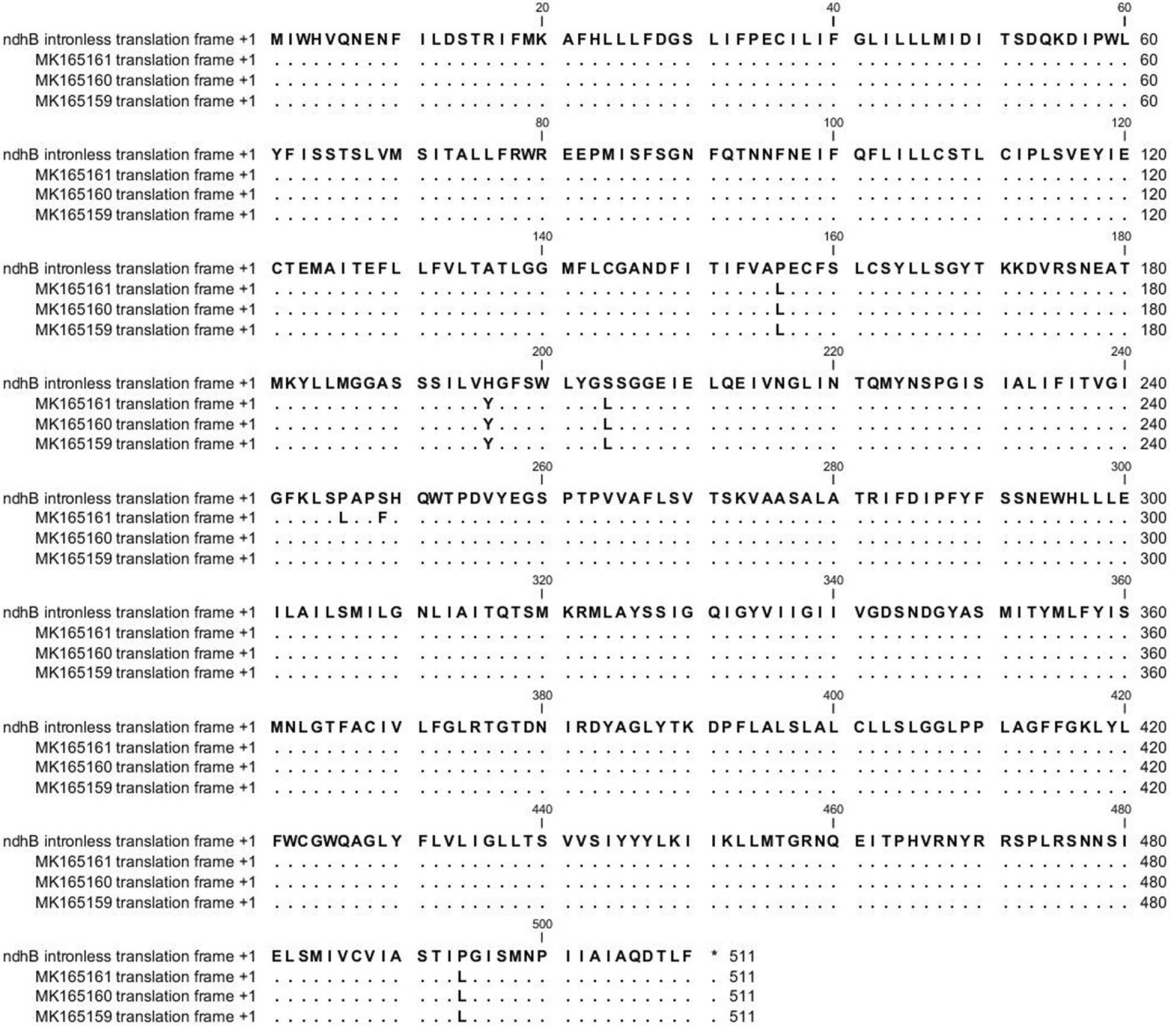
Effect of RNA editing at protein level. Comparison of C. procera ndhB protein deduced from intronless ndhB DNA and ndhB proteins deduced from three times cDNA sequences

A comparison of genomic *ndhB* sequences with 3-times cDNA (Fig. S2) showed conversion of C to U in 7 RNA positions (nucleotide no. C467, C586, C611, C737, C746, C830, and C1481). Since the rate editing is less than 50% in all transcripts (Fig. 1, Table S1), there was another editing site in C830 that does not appear in Fig. S2. The previous RNA editing resulted in the substitution of 7 amino acids: (i) three proline to leucine (P-L), (ii) two serine to leucine (S-L), (iii) one serine to phenylalanine (S-F), and (iv) one histidine to tyrosine (H-Y) (Fig. 1, Fig. S2, Table S1).

### Analysis of the deduced protein sequence and conserved domain

**Fig. 2:**
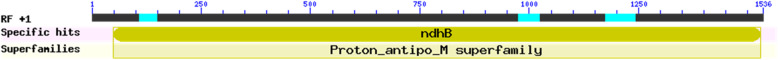
Protein domains of the deduced amino acid sequence of the obtained ndhB protein (RF)

**Fig. 3:**
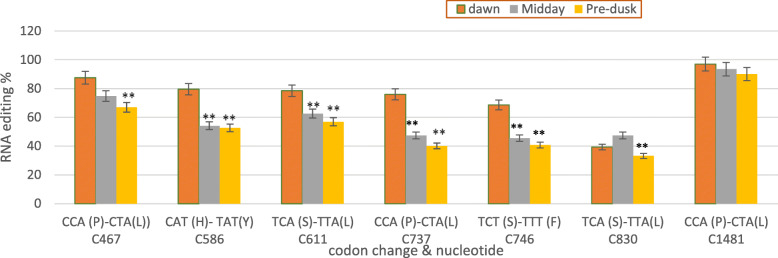
Differential and efficiency of nad RNA editing, comparing with dawn, using reads derived from total RNA-seq. (P) Proline, (L) Leucine, (H) Histidine, (Y) Tyrosine, (S) Serine, (F) Phenylalanine. Data are expressed as means with ±SD (black bars) of three biological replicates. ** indicate significant difference between treatments *P* < 0.01.

The current effect of editing the RNA must be measured at the level of protein. The sequences of amino acids derived from *C. procera* genomic and complementary DNA (cDNA) were compared with the amino acid profile of other species derived from cDNA. This comparison clearly demonstrated that the editing in *ndhB* gene in *C. procera* led to the formation of conserved amino acid (Fig. 2). Domain analysis refers to the activity of chloroplast NADH-plastoquinone oxidoreductase subunit 2 (*ndhB*) and CDD accession number cl00535, and Pfam, PF00507 (Fig. 3).

### Validation of editing in *ndhB* gene

**Fig. 4:**
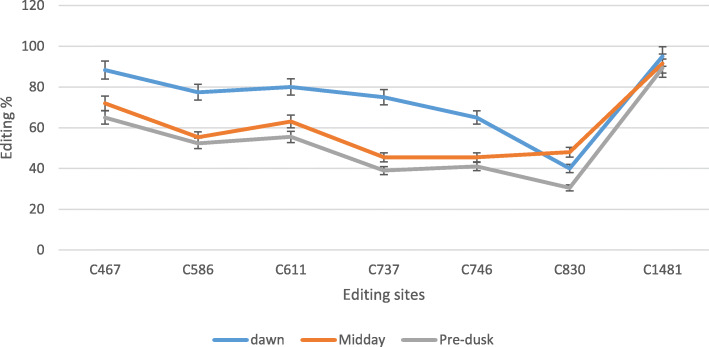
QRT-PCR confirmation of *Calotropis procera* ndhB editing sites predicted by CLC genomic workbench in different 3 times (dawn, midday, and pre-dusk). Data are expressed as means with ± SD (black bars) of three biological replicates

To validate candidate editing sites, and clarify the importance of RNA-seq tool in RNA editing identification, the seven editing sites were analyzed using RT-qPCR. The *ndhB* editing positions (C467, C586, C611, C737, C746, 830, and C1481) were comparatively quantified in three light periods (Fig. 4). RT-qPCR data confirmed that the editing percentage was the highest at dawn except in C830 which was higher at midday. Data were analyzed using CLC genomic workbench version 3.6.5.

### 3D structure alignment

**Fig. 5:**
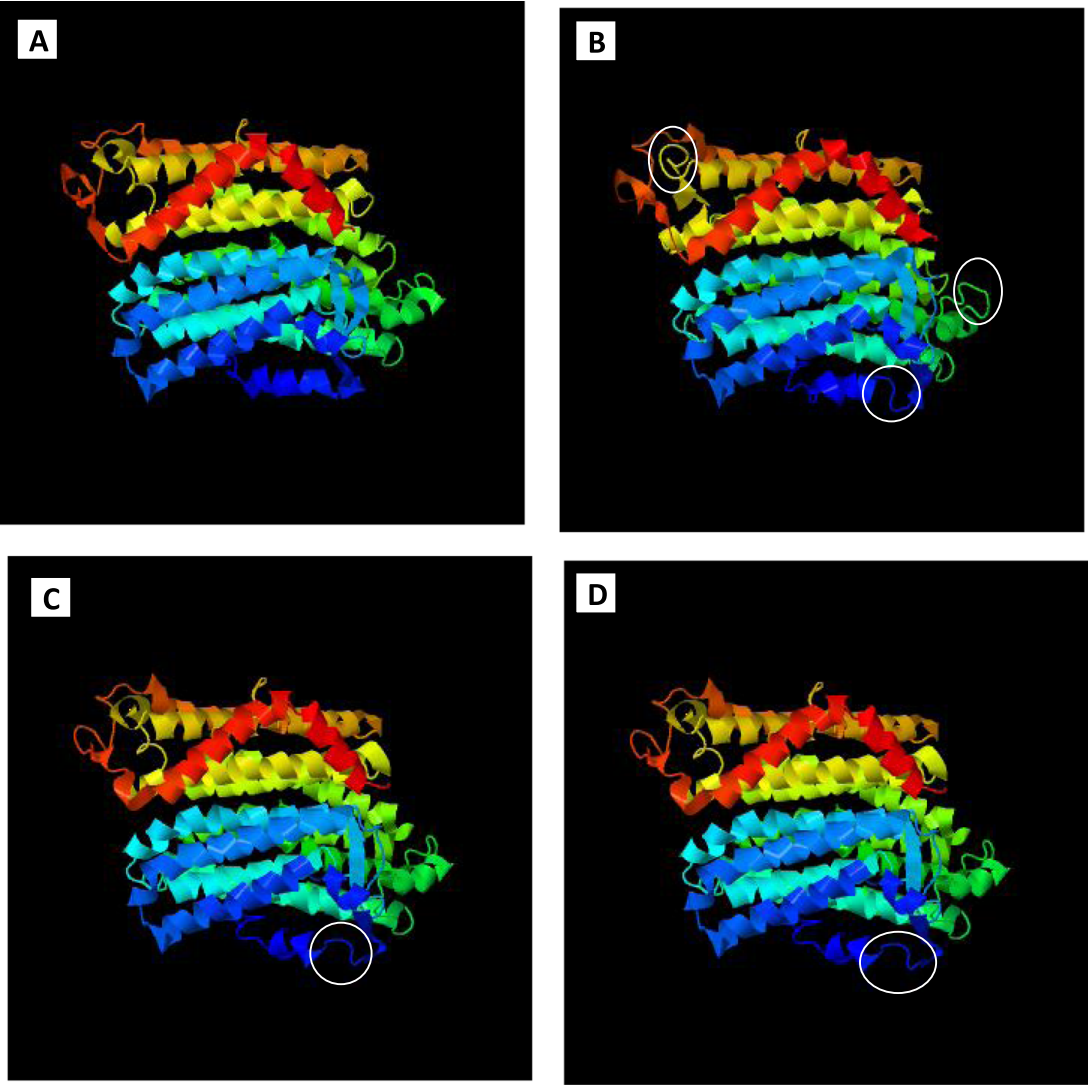
3D model of C*. procera ndhB* protein in different three times as well as original protein. **a** Predicted 3D protein of correspondence DNA coding sequence. **b** Predicted 3D protein at dawn. **c** Predicted 3D protein at midday. **d** Predicted 3D protein at pre-dusk, white circle showed the differences in loop domains occurred from RNA editing

3D models of three times *ndhB* proteins structure as well as correspondence protein of DNA coding sequence are compared. The result showed changes in three loops region in *ndhB* protein at dawn, and one at midday; however, the protein at pre-dusk was identical to midday in shape (Fig. 5).

## Discussion

RNA editing is common in terrestrial plants, especially in the mitochondria [5, 12, 27] and chloroplast [7, 28]. It plays a crucial role in the expression of functional proteins, like RNA editing *in Nicotiana tomentosiformis* ndhD-1 site which restore a translational initiation codon ATG from ACG on its DNA regulates the translation efficiency [29]. But, the lower level of RNA editing at some sites does not affect in accumulation of chloroplast NDH complex in *Nicotiana tomentosiformis* [30]. Otherwise, the disturbance of C250 editing in mitochondrial Nad dehydrogenase subunit 3 (nad3) gene results in the accumulation of large reactive oxygen species (ROS) concentrations, leading to a deterioration of *Arabidopsis* drought tolerance [31].

RNA editing in *ndhB* gene was reported since three decades [32]. RNA editing in *ndhB* gene was detected at 9 sites in *Arabidopsis* (*Arabidopsis thaliana*), 10 in rice, 13 in soybean [7, 25], and 8 in *Crambe* (*Crambe abyssinica*) [33]. In this study, seven editing sites of *C. procera ndhB* gene (C467, C586, C611, C737, C746, C830, and C1481) were deferentially edited across three daylight periods. These sites were validated by qRT-PCR. The identified edited sites in *C. procera* were concordant with previous research achieved by [25]. However, there were five other editing sites (C836, C1112, C1255, C1391, and C1414) reported by the same study [25], and they were not found in *ndhB* transcript in *C. procera*. Most of the non-editing sites were unchanged because they were T, not C, in the target gene. Furthermore, RNA editing rate of *ndhB* gene was highest at dawn, (87.5, 79.6, 78.5, 76, 68.6, 39.3, and 96.9%, respectively), less at midday (74.8, 54.1, 62.6, 47.4, 45.5, 47.4, and 93.4%, respectively), and lowest at pre-dusk (67, 52.6, 56.9, 40.1, 40.7, 33.2, and 90%, respectively). RNA editing can modify hydrophilic amino acids to hydrophobic amino acids and occur at locations necessary for 3D protein structure formation and protein folding [34]. The produced 3D structure of three times ndhB protein showed differential structure in dawn and midday due to differential in RNA editing. This indicate to the protein function and activity is affected by RNA editing as reported in previous studies [35, 36].

These results indicated that light period may affect the rate of RNA editing without induction of new editing sites. From current results, we do speculate that photosynthesis in this desert plant may reach its maximum during dawn daylight time. In fact, RNA editing may increase hydrophobicity of protein and its affinity to phospholipids in the chloroplast’s membrane [35, 36]. However, such hypothesis needs to be investigated and it will be the subject of our future studies.

## Conclusion

In *nhdB* transcript in *C. procera*, comprehensive editing takes place at 7 sites; these editing sites were primarily conserved through plant species. The differential editing of this gene across light periods may be led to a somehow relations between the RNA editing and control of photosynthesis. The main limitation of the study was the lack of understanding of how the light may control the level of RNA editing in the chloroplast in *C. procera*, which will be the main focus of future research taking into consideration circadian changes can occur.

## Supplementary information

**Additional file 1: **Table S1. *NdhB* C-to-U editing events using reads derived from total RNA-seq.

**Additional file 2:.** Fig. S1 Multi-sequence alignment was enforced using the best hits of BLAST search.

**Additional file 3: **Fig S2. A comparison between *ndhB* sequences of the genomic DNA and 3 times cDNA revealed RNA editing sites.

## Data Availability

All data generated or analyzed during this study are included in this published article [and its supplementary information files].

## Abbreviations

Nad: Nad dehydreogenase
Cytb: Cytochrome b reductase
COX: Cytochrome c oxidase
apobec: Apolipoprotein-B catalytic subunit
ACF: Apobec-1 complementation factor
*atp1*: Atp synthase subunit 1
*nad3*: Nad dehydreogenase gene subunit 3
*nad5*: Nad dehydreogenase gene subunit 5
*rpl2*: Ribosomal *protein* L2
*rps1*: Ribosomal *protein* S1
