# Supplementary material for: Light/heat effects on RNA editing in chloroplast NADH-plastoquinone oxidoreductase subunit 2 (ndhB) gene of Calotropis (Calotropis procera)
Source: J Genet Eng Biotechnol. 2020 Sep 11;18:49. doi: 10.1186/s43141-020-00064-4 (PMC7486354; doi:10.1186/s43141-020-00064-4)
Supplement: Supplementary file 1 — Additional file 1: Table S1. NdhB C-to-U editing events using reads derived from total RNA-seq. [file 43141_2020_64_MOESM1_ESM.docx]

Table (S1). NdhB C-to-U editing events using reads derived from total RNA-seq.

| **Light period** | **Codon change** | **Nucleotide position** | **AA change** | **AA position** | **Total coverage** | **Edited coverage** | **% Editing** |
| --- | --- | --- | --- | --- | --- | --- | --- |
| dawn | CCA (P)-CTA(L) | 467 | P- L | 156 | 327 | 286 | 87.5 |
|  | CAT (H)- TAT(Y) | 586 | H- Y | 196 | 678 | 540 | 79.6 |
|  | TCA (S)-TTA(L) | 611 | S- L | 204 | 1013 | 795 | 78.5 |
|  | CCA (P)-CTA(L) | 737 | P- L | 246 | 1827 | 1388 | 76 |
|  | TCT (S)-TTT (F) | 746 | S- F | 249 | 1450 | 994 | 68.6 |
|  | TCA (S)-TTA(L) | 830 | S- L | 277 | 900 | 358 | 39.3 |
|  | CCA (P)-CTA(L) | 1481 | P- L | 494 | 1034 | 1067 | 96.9 |
| Midday | CCA (P)-CTA(L) | 467 | P- L | 156 | 127 | 95 | 74.8 |
|  | CAT (H)- TAT(Y) | 586 | H- Y | 196 | 231 | 125 | 54.1 |
|  | TCA (S)-TTA(L) | 611 | S- L | 204 | 313 | 196 | 62.6 |
|  | CCA (P)-CTA(L) | 737 | P- L | 246 | 599 | 284 | 47.4 |
|  | TCT (S)-TTT (F) | 746 | S- F | 249 | 466 | 212 | 45.5 |
|  | TCA- TTA | 830 | S- L | 277 | 285 | 135 | 47.4 |
|  | CCA (P)-CTA(L) | 1481 | P- L | 494 | 361 | 337 | 93.4 |
| Pre-dusk | CCA (P)-CTA(L) | 467 | P- L | 156 | 91 | 61 | 67 |
|  | CAT (H)- TAT(Y) | 586 | H- Y | 196 | 213 | 112 | 52.6 |
|  | TCA (S)-TTA(L) | 611 | S- L | 204 | 346 | 197 | 56.9 |
|  | CCA (P)-CTA(L) | 737 | P- L | 246 | 741 | 297 | 40.1 |
|  | TCT (S)-TTT (F) | 746 | S- F | 249 | 619 | 252 | 40.7 |
|  | TCA- TTA | 830 | S- L | 277 | 262 | 87 | 33.2 |
|  | CCA (P)-CTA(L) | 1481 | P- L | 494 | 418 | 376 | 90 |
