# Supplementary material for: Light/heat effects on RNA editing in chloroplast NADH-plastoquinone oxidoreductase subunit 2 (ndhB) gene of Calotropis (Calotropis procera)
Source: J Genet Eng Biotechnol. 2020 Sep 11;18:49. doi: 10.1186/s43141-020-00064-4 (PMC7486354; doi:10.1186/s43141-020-00064-4)
Supplement: Supplementary file 2 — Additional file 2:. Fig. S1 Multi-sequence alignment was enforced using the best hits of BLAST search. [file 43141_2020_64_MOESM2_ESM.pdf]

```
                                                    20                             40                             60
                                                     |                              |                              |
                    MK144329  ATGATCTGGC  ATGTACAGAA  TGAAAACTTC  ATTCTCGATT  CTACGAGAAT  TTTTATGAAA  60
MH939982.1:c106394-104178  ..........  ..........  ..........  ..........  ..........  ..........  60
MH939981.1:c106661-104445  ..........  ..........  ..........  ..........  ..........  ..........  60
  KF539850.1:c100173-97957  ..........  ..........  ..........  ..........  ..........  ..........  60
MG678915.1:c103141-100925  ..........  ..........  ..........  ..........  ..........  ..........  60
MG678876.1:c103509-101293  ..........  ..........  ..........  ..........  ..........  ..........  60
MG678856.1:c103368-101152  ..........  ..........  ..........  ..........  ..........  ..........  60
MG678843.1:c103267-101051  ..........  ..........  ..........  ..........  ..........  ..........  60
  MG678835.1:c100962-98746  ..........  ..........  ..........  ..........  ..........  ..........  60
    KF539853.1:c98719-96503  ..........  ..........  ..........  ..........  ..........  ..........  60
    KF539852.1:c99164-96948  ..........  ..........  ..........  ..........  ..........  ..........  60
                                                    80                            100                            120
                                                     |                              |                              |
                    MK144329  GCCTTTCATT  TGCTTCTCTT  CGATGGAAGT  TTGATTTTCC  CAGAATGTAT  CCTAATTTTT  120
MH939982.1:c106394-104178  ..........  ..........  ..........  ..........  ..........  ..........  120
MH939981.1:c106661-104445  ..........  ..........  ..........  ..........  ..........  ..........  120
  KF539850.1:c100173-97957  ..........  ..........  ..........  ..........  ..........  ..........  120
MG678915.1:c103141-100925  ..........  ..........  ..........  ..........  ..........  ..........  120
MG678876.1:c103509-101293  ..........  ..........  ..........  ..........  ..........  ..........  120
MG678856.1:c103368-101152  ..........  ..........  ..........  ..........  ..........  ..........  120
MG678843.1:c103267-101051  ..........  ..........  ..........  ..........  ..........  ..........  120
  MG678835.1:c100962-98746  ..........  ..........  ..........  ..........  ..........  ..........  120
    KF539853.1:c98719-96503  ..........  ..........  ..........  ..........  ..........  ..........  120
    KF539852.1:c99164-96948  ..........  ..........  ..........  ..........  ..........  ..........  120
                                                   140                            160                            180
                                                     |                              |                              |
                    MK144329  GGCCTAATTC  TTCTTTTGAT  GATCGATATA  ACCTCGGATC  AAAAAGATAT  ACCTTGGTTA  180
MH939982.1:c106394-104178  ..........  ..........  ..........  ..........  ..........  ..........  180
MH939981.1:c106661-104445  ..........  ..........  ..........  ..........  ..........  ..........  180
  KF539850.1:c100173-97957  ..........  ..........  ..........  ..........  ..........  ..........  180
MG678915.1:c103141-100925  ..........  ..........  ..........  ..........  ..........  ..........  180
MG678876.1:c103509-101293  ..........  ..........  ..........  ..........  ..........  ..........  180
MG678856.1:c103368-101152  ..........  ..........  ..........  ..........  ..........  ..........  180
MG678843.1:c103267-101051  ..........  ..........  ..........  ..........  ..........  ..........  180
  MG678835.1:c100962-98746  ..........  ..........  ..........  ..........  ..........  ..........  180
    KF539853.1:c98719-96503  ..........  ..........  ..........  ..........  ..........  ..........  180
    KF539852.1:c99164-96948  ..........  ..........  .......C..  ..........  ..........  ..........  180
                                                   200                            220                            240
                                                     |                              |                              |
                    MK144329  TATTTCATCT  CTTCAACAAG  TTTAGTAATG  AGCATAACGG  CCCTATTGTT  CCGATGGAGA  240
MH939982.1:c106394-104178  ..........  ..........  ..........  ..........  ..........  ..........  240
MH939981.1:c106661-104445  ..........  ..........  ..........  ..........  ..........  ..........  240
  KF539850.1:c100173-97957  ..........  ..........  ..........  ..........  ..........  ..........  240
MG678915.1:c103141-100925  ..........  ..........  ..........  ..........  ..........  ..........  240
MG678876.1:c103509-101293  ..........  ..........  ..........  ..........  ..........  ..........  240
MG678856.1:c103368-101152  ..........  ..........  ..........  ..........  ..........  ..........  240
MG678843.1:c103267-101051  ..........  ..........  ..........  ..........  ..........  ..........  240
  MG678835.1:c100962-98746  ..........  ..........  ..........  ..........  ..........  ..........  240
    KF539853.1:c98719-96503  ..........  ..........  ..........  ..........  ..........  ..........  240
    KF539852.1:c99164-96948  ..........  ..........  ..........  ..........  ..........  ..........  240
                                                   260                            280                            300
                                                     |                              |                              |
                    MK144329  GAAGAACCTA  TGATTAGCTT  TTCGGGAAAT  TTCCAAACGA  ACAATTTCAA  CGAAATCTTT  300
MH939982.1:c106394-104178  ..........  ..........  ..........  ..........  ..........  ..........  300
MH939981.1:c106661-104445  ..........  ..........  ..........  ..........  ..........  ..........  300
  KF539850.1:c100173-97957  ..........  ..........  ..........  ..........  ..........  ..........  300
MG678915.1:c103141-100925  ..........  ..........  ..........  ..........  ..........  ..........  300
MG678876.1:c103509-101293  ..........  ..........  ..........  ..........  ..........  ..........  300
MG678856.1:c103368-101152  ..........  ..........  ..........  ..........  ..........  ..........  300
MG678843.1:c103267-101051  ..........  ..........  ..........  ..........  ..........  ..........  300
  MG678835.1:c100962-98746  ..........  ..........  ..........  ..........  ..........  ..........  300
    KF539853.1:c98719-96503  ..........  ..........  ..........  ..........  ..........  ..........  300
    KF539852.1:c99164-96948  ..........  ..........  ..........  ..........  ..........  ..........  300
                                                   320                            340                            360
                                                     |                              |                              |
                    MK144329  CAATTTCTTA  TTTTACTATG  TTCAACTCTA  TGTATTCCTC  TATCCGTAGA  GTACATTGAA  360
MH939982.1:c106394-104178  ..........  ..........  ..........  ..........  ..........  ..........  360
MH939981.1:c106661-104445  ..........  ..........  ..........  ..........  ..........  ..........  360
  KF539850.1:c100173-97957  ..........  ..........  ..........  ..........  ..........  ..........  360
MG678915.1:c103141-100925  ..........  ..........  ..........  ..........  ..........  ..........  360
MG678876.1:c103509-101293  ..........  ..........  ..........  ..........  ..........  ..........  360
MG678856.1:c103368-101152  ..........  ..........  ..........  ..........  ..........  ..........  360
MG678843.1:c103267-101051  ..........  ..........  ..........  ..........  ..........  ..........  360
  MG678835.1:c100962-98746  ..........  ..........  ..........  ..........  ..........  ..........  360
    KF539853.1:c98719-96503  ..........  ..........  ..........  ..........  ..........  ..........  360
    KF539852.1:c99164-96948  ..........  ..........  ..........  ..........  ..........  ..........  360
```

```
                                                             380                            400                            420
                                                               |                              |                              |
MK144329                    TGTACAGAAA TGGCTATAAC AGAGTTTCTC TTATTCGTAT TAACAGCTAC TCTGGGAGGA 420
MH939982.1:c106394-104178   .......... .......... .......... .......... .......... .......... 420
MH939981.1:c106661-104445   .......... .......... .......... .......... .......... .......... 420
KF539850.1:c100173-97957    .......... .......... .......... .......... .......... .......... 420
MG678915.1:c103141-100925   .......... .......... .......... .......... .......... .......... 420
MG678876.1:c103509-101293   .......... .......... .......... .......... .......... .......... 420
MG678856.1:c103368-101152   .......... .......... .......... .......... .......... .......... 420
MG678843.1:c103267-101051   .......... .......... .......... .......... .......... .......... 420
MG678835.1:c100962-98746    .......... .......... .......... .......... .......... .......... 420
KF539853.1:c98719-96503     .......... .......... .......... .......... .......... .......... 420
KF539852.1:c99164-96948     .......... .......... .......... .......... .......... .......... 420
                                                             440                            460                            480
                                                               |                              |                              |
MK144329                    ATGTTTTTAT GCGGTGCTAA CGATTTCATA ACTATCTTTG TAGCTCCAGA ATGTTTCAGT 480
MH939982.1:c106394-104178   .......... .......... .......... .......... .......... .......... 480
MH939981.1:c106661-104445   .......... .......... .......... .......... .......... .......... 480
KF539850.1:c100173-97957    .......... .......... .......... .......... .......... .......... 480
MG678915.1:c103141-100925   .......... .......... .......... .......... .......... .......... 480
MG678876.1:c103509-101293   .......... .......... .......... .......... .......... .......... 480
MG678856.1:c103368-101152   .......... .......... .......... .......... .......... .......... 480
MG678843.1:c103267-101051   .......... .......... .......... .......... .......... .......... 480
MG678835.1:c100962-98746    .......... .......... .......... .......... .......... .......... 480
KF539853.1:c98719-96503     .......... .......... .......... .......... .......... .......... 480
KF539852.1:c99164-96948     .......... .......... .......... .......... .......... .......... 480
                                                             500                            520                            540
                                                               |                              |                              |
MK144329                    TTATGTTCCT ACCTATTATC TGGATATACC AAGAAAGATG TACGGTCTAA TGAGGCTACT 540
MH939982.1:c106394-104178   .......... .......... .......... .......... .......... .......... 540
MH939981.1:c106661-104445   .......... .......... .......... .......... .......... .......... 540
KF539850.1:c100173-97957    .......... .......... .......... .......... .......... .......... 540
MG678915.1:c103141-100925   .......... .......... .......... .......... .......... .......... 540
MG678876.1:c103509-101293   .......... .......... .......... .......... .......... .......... 540
MG678856.1:c103368-101152   .......... .......... .......... .......... .......... .......... 540
MG678843.1:c103267-101051   .......... .......... .......... .......... .......... .......... 540
MG678835.1:c100962-98746    .......... .......... .......... .......... .......... .......... 540
KF539853.1:c98719-96503     .......... .......... .......... .......... .......... .......... 540
KF539852.1:c99164-96948     .......... .......... .......... .......... .......... .......... 540
                                                             560                            580                            600
                                                               |                              |                              |
MK144329                    ATGAAATATT TACTCATGGG TGGGGCAAGC TCTTCTATTC TGGTTCATGG TTTCTCTTGG 600
MH939982.1:c106394-104178   .......... .......... .......... .......... .......... .......... 600
MH939981.1:c106661-104445   .......... .......... .......... .......... .......... .......... 600
KF539850.1:c100173-97957    .......... .......... .......... .......... .......... .......... 600
MG678915.1:c103141-100925   .......... .......... .......... .......... .......... .......... 600
MG678876.1:c103509-101293   .......... .......... .......... .......... .......... .......... 600
MG678856.1:c103368-101152   .......... .......... .......... .......... .......... .......... 600
MG678843.1:c103267-101051   .......... .......... .......... .......... .......... .......... 600
MG678835.1:c100962-98746    .......... .......... .......... .......... .......... .......... 600
KF539853.1:c98719-96503     .......... .......... .......... .......... .......... .......... 600
KF539852.1:c99164-96948     .......... .......... .......... .......... .......... .......... 600
                                                             620                            640                            660
                                                               |                              |                              |
MK144329                    CTATATGGTT CATCCGGGGG AGAGATCGAG CTTCAAGAAA TAGTGAATGG TCTTATCAAT 660
MH939982.1:c106394-104178   .......... .......... .......... .......... .......... .......... 660
MH939981.1:c106661-104445   .......... .......... .......... .......... .......... .......... 660
KF539850.1:c100173-97957    .......... .......... .......... .......... .......... .......... 660
MG678915.1:c103141-100925   .......... .......... .......... .......... .......... .......... 660
MG678876.1:c103509-101293   .......... .......... .......... .......... .......... .......... 660
MG678856.1:c103368-101152   .......... .......... .......... .......... .......... .......... 660
MG678843.1:c103267-101051   .......... .......... .......... .......... .......... .......... 660
MG678835.1:c100962-98746    .......... .......... .......... .......... .......... .......... 660
KF539853.1:c98719-96503     .......... .......... .......... .......... .......... .......... 660
KF539852.1:c99164-96948     .......... .......... .......... .......... .......... .......... 660
                                                             680                            700                            720
                                                               |                              |                              |
MK144329                    ACACAAATGT ATAACTCCCC AGGAATTTCA ATTGCGCTCA TATTCATTAC TGTAGGAATT 720
MH939982.1:c106394-104178   .......... .......... .......... .......... .......... .......... 720
MH939981.1:c106661-104445   .......... .......... .......... .......... .......... .......... 720
KF539850.1:c100173-97957    .......... .......... .......... .......... .......... .......... 720
MG678915.1:c103141-100925   .......... .......... .......... .......... .......... .......... 720
MG678876.1:c103509-101293   .......... .......... .......... .C........ .......... .......... 720
MG678856.1:c103368-101152   .......... .......... .......... .C........ .......... .......... 720
MG678843.1:c103267-101051   .......... .......... .......... .C........ .......... .......... 720
MG678835.1:c100962-98746    .......... .......... .......... .C........ .......... .......... 720
KF539853.1:c98719-96503     .......... .......... .......... .......... .......... .......... 720
KF539852.1:c99164-96948     .......... .......... .......... .......... .......... .......... 720
```

```
                                                 740                           760                           780
                                                   |                             |                             |
MK144329                   GGGTTCAAGC TTTCCCCAGC CCCTTCTCAT CAATGGACTC CTGACGTATA CGAAGGAGTG 780
MH939982.1:c106394-104178  .......... .......... .......... .......... .......... .......... 780
MH939981.1:c106661-104445  .......... .......... .......... .......... .......... .......... 780
KF539850.1:c100173-97957   .......... .......... .......... .......... .......... .......... 780
MG678915.1:c103141-100925  .......... .......... .......... .......... .......... .......... 780
MG678876.1:c103509-101293  .......... .......... .......... .......... .......... .......... 780
MG678856.1:c103368-101152  .......... .......... .......... .......... .......... .......... 780
MG678843.1:c103267-101051  .......... .......... .......... .......... .......... .......... 780
MG678835.1:c100962-98746   .......... .......... .......... .......... .......... .......... 780
KF539853.1:c98719-96503    .......... .......... .......... .......... .......... .......... 780
KF539852.1:c99164-96948    .......... .......... .......... .......... .......... .......... 780
                                                 800                           820                           840
                                                   |                             |                             |
MK144329                   CGGTTCGTTC GAGAAATTCC TACCTCTCTA TCTATCTCTA AGATGTTTGG ATTTTTCAAA 840
MH939982.1:c106394-104178  .......... .......... .......... .......... .......... .......... 840
MH939981.1:c106661-104445  .......... .......... .......... .......... .......... .......... 840
KF539850.1:c100173-97957   .......... .......... .......... .......... .......... .......... 840
MG678915.1:c103141-100925  .......... .......... .......... .......... .......... .......... 840
MG678876.1:c103509-101293  .......... .......... .......... .......... .......... .......... 840
MG678856.1:c103368-101152  .......... .......... .......... .......... .......... .......... 840
MG678843.1:c103267-101051  .......... .......... .......... .......... .......... .......... 840
MG678835.1:c100962-98746   .......... .......... .......... .......... .......... .......... 840
KF539853.1:c98719-96503    .......... .......... .......... .......... .......... .......... 840
KF539852.1:c99164-96948    .......... .......... .......... .......... .......... .......... 840
                                                 860                           880                           900
                                                   |                             |                             |
MK144329                   ACTCCATGGA CATGCAGAAG AGAAATGCTA TCCCCACTCG GACCAAGACA GAACTTTTAC 900
MH939982.1:c106394-104178  .......... .......... .......... .......... .......... .......... 900
MH939981.1:c106661-104445  .......... .......... .......... .......... .......... .......... 900
KF539850.1:c100173-97957   .......... .......... .......... .......... .......... .......... 900
MG678915.1:c103141-100925  .......... .......... .......... .......... .......... .......... 900
MG678876.1:c103509-101293  .......... .......... .......... .......... .......... .......... 900
MG678856.1:c103368-101152  .......... .......... .......... .......... .......... .......... 900
MG678843.1:c103267-101051  .......... .......... .......... .......... .......... .......... 900
MG678835.1:c100962-98746   .......... .......... .......... .......... .......... .......... 900
KF539853.1:c98719-96503    .......... .......... .......... .......... .......... .......... 900
KF539852.1:c99164-96948    .......... .......... .......... .......... .......... .......... 900
                                                 920                           940                           960
                                                   |                             |                             |
MK144329                   TTGTTCAAAT AACAATTAAG GTGAAGCGGG GTCAAGAACG ACGAATCTCT TTATGATAAA 960
MH939982.1:c106394-104178  .......... .......... .......... .......... .......... .......... 960
MH939981.1:c106661-104445  .......... .......... .......... .......... .......... .......... 960
KF539850.1:c100173-97957   .......... .......... .......... .......... .......... .......... 960
MG678915.1:c103141-100925  .......... .......... .......... .......... .......... .......... 960
MG678876.1:c103509-101293  .......... .......... .......... .......... .......... .......... 960
MG678856.1:c103368-101152  .......... .......... .......... .......... .......... .......... 960
MG678843.1:c103267-101051  .......... .......... .......... .......... .......... .......... 960
MG678835.1:c100962-98746   .......... .......... .......... .......... .......... .......... 960
KF539853.1:c98719-96503    .......... .......... .......... .......... .......... .......... 960
KF539852.1:c99164-96948    .......... .......... .......... .......... .......... .......... 960
                                                 980                         1,000                         1,020
                                                   |                             |                             |
MK144329                   CAGATCCATT TTGCAAGTTC CTTATTACGG GTAGTTCCTA CAAAGGATCG GACTAATGAC 1020
MH939982.1:c106394-104178  .......... .......... .......... .......... .......... .......... 1020
MH939981.1:c106661-104445  .......... .......... .......... .......... .......... .......... 1020
KF539850.1:c100173-97957   .......... .......... .......... .......... .......... .......... 1020
MG678915.1:c103141-100925  .......... .......... .......... .......... .......... .......... 1020
MG678876.1:c103509-101293  .......... .......... .......... .......... .......... .......... 1020
MG678856.1:c103368-101152  .......... .......... .......... .......... .......... .......... 1020
MG678843.1:c103267-101051  .......... .......... .......... .......... .......... .......... 1020
MG678835.1:c100962-98746   .......... .......... .......... .......... .......... .......... 1020
KF539853.1:c98719-96503    .......... .......... .......... .......... .......... .......... 1020
KF539852.1:c99164-96948    .......... .......... .......... .......... .......... .......... 1020
                                               1,040                         1,060                         1,080
                                                   |                             |                             |
MK144329                   GTATACAATA CTTGAATTCT CGATGTAGAT GCTACATAGT TGGTTCTCGT CCTTCAGAGA 1080
MH939982.1:c106394-104178  .......... .......... .......... .......... .......... .......... 1080
MH939981.1:c106661-104445  .......... .......... .......... .......... .......... .......... 1080
KF539850.1:c100173-97957   .......... .......... .......... .......... .......... .......... 1080
MG678915.1:c103141-100925  .......... .......... .......... .......... .......... .......... 1080
MG678876.1:c103509-101293  .......... .......... .......... .......... .......... .......... 1080
MG678856.1:c103368-101152  .......... .......... .......... .......... .......... .......... 1080
MG678843.1:c103267-101051  .......... .......... .......... .......... .......... .......... 1080
MG678835.1:c100962-98746   .......... .......... .......... .......... .......... .......... 1080
KF539853.1:c98719-96503    .......... .......... .......... .......... .......... .......... 1080
KF539852.1:c99164-96948    .......... .......... .......... .......... .......... .......... 1080
```

```
                                                   1,100                1,120                1,140
MK144329                   CTACGAGTGT AATAAGAGCA TCCGTCGACA AAAGGATCAC CCTAAGATGA TCATCTCATG 1140
MH939982.1:c106394-104178  .......... .......... .......... .......... .......... .......... 1140
MH939981.1:c106661-104445  .......... .......... .......... .......... .......... .......... 1140
KF539850.1:c100173-97957   .......... .......... .......... .......... .......... .......... 1140
MG678915.1:c103141-100925  .......... .......... .......... .......... .......... .......... 1140
MG678876.1:c103509-101293  .......... .......... .......... .......... .......... .......... 1140
MG678856.1:c103368-101152  .......... .......... .......... .......... .......... .......... 1140
MG678843.1:c103267-101051  .......... .......... .......... .......... .......... .......... 1140
MG678835.1:c100962-98746   .......... .......... .......... .......... .......... .......... 1140
KF539853.1:c98719-96503    .......... .......... .......... .......... .......... .......... 1140
KF539852.1:c99164-96948    .......... .......... .......... .......... .......... .......... 1140
                                                   1,160                1,180                1,200
MK144329                   GCTATTGAGA ACGAATTAAA TCAGATGGTT CTATTTCTCA ATCTTTCTGA CTTGCTTTGC 1200
MH939982.1:c106394-104178  .......... .......... .......... .......... .......... .......... 1200
MH939981.1:c106661-104445  .......... .......... .......... .......... .......... .......... 1200
KF539850.1:c100173-97957   .......... .......... .......... .......... .......... .......... 1200
MG678915.1:c103141-100925  .......... .......... .......... .......... .......... .......... 1200
MG678876.1:c103509-101293  .......... .......... .......... .......... .......... .......... 1200
MG678856.1:c103368-101152  .......... .......... .......... .......... .......... .......... 1200
MG678843.1:c103267-101051  .......... .......... .......... .......... .......... .......... 1200
MG678835.1:c100962-98746   .......... .......... .......... .......... .......... .......... 1200
KF539853.1:c98719-96503    .......... .......... .......... .......... ...G...... .......... 1200
KF539852.1:c99164-96948    .......... .......... .......... .......... .......... .......... 1200
                                                   1,220                1,240                1,260
MK144329                   TCCTACGGAA CCAAGGTCGA AAGGATTGAA AAAATCAGTC ATTCACAACC ACTGATGAAG 1260
MH939982.1:c106394-104178  .......... .......... .......... .......... .......... .......... 1260
MH939981.1:c106661-104445  .......... .......... .......... .......... .......... .......... 1260
KF539850.1:c100173-97957   .......... .......... .......... .......... .......... .......... 1260
MG678915.1:c103141-100925  .......... .......... .......... .......... .......... .......... 1260
MG678876.1:c103509-101293  .......... .......... .......... .......... .......... .......... 1260
MG678856.1:c103368-101152  .......... .......... .......... .......... .......... .......... 1260
MG678843.1:c103267-101051  .......... .......... .......... .......... .......... .......... 1260
MG678835.1:c100962-98746   .......... .......... .......... .......... .......... .......... 1260
KF539853.1:c98719-96503    .......... .......... .......... .......... .......... .......... 1260
KF539852.1:c99164-96948    .......... .......... .......... .......... .......... .......... 1260
                                                   1,280                1,300                1,320
MK144329                   GATTCCTCGA AAAGTTAAGG ATTAGTAATC CTTTTTAGAA ATCGAATGGA TTCGGTCTTA 1320
MH939982.1:c106394-104178  .......... .......... .......... .......... .......... .......... 1320
MH939981.1:c106661-104445  .......... .......... .......... .......... .......... .......... 1320
KF539850.1:c100173-97957   .......... .......... .......... .......... .......... .......... 1320
MG678915.1:c103141-100925  .......... .......... ....C..... .......... .......... .......... 1320
MG678876.1:c103509-101293  .......... .......... .......... .......... .......... .......... 1320
MG678856.1:c103368-101152  .......... .......... .......... .......... .......... .......... 1320
MG678843.1:c103267-101051  .......... .......... .......... .......... .......... .......... 1320
MG678835.1:c100962-98746   .......... .......... .......... .......... .......... .......... 1320
KF539853.1:c98719-96503    .......... .......... .......... .......... .......... .......... 1320
KF539852.1:c99164-96948    .......... .......... .......... .......... .......... .......... 1320
                                                   1,340                1,360                1,380
MK144329                   TACATACGCG AGGAAGGTAA TCAAAAAAGA AAGAAGATGA GTTCTTCTTT CTTTTATCAC 1380
MH939982.1:c106394-104178  .......... .......... .......... .......... .......... .......... 1380
MH939981.1:c106661-104445  .......... .......... .......... .......... .......... .......... 1380
KF539850.1:c100173-97957   .......... .......... .......... .......... .......... .......... 1380
MG678915.1:c103141-100925  .......... .......... .......... .......... .......... .......... 1380
MG678876.1:c103509-101293  .......... .......... .......... .......... .......... .......... 1380
MG678856.1:c103368-101152  .......... .......... .......... .......... .......... .......... 1380
MG678843.1:c103267-101051  .......... .......... .......... .......... .......... .......... 1380
MG678835.1:c100962-98746   .......... .......... .......... .......... .......... .......... 1380
KF539853.1:c98719-96503    .......... .......... .......... .......... .......... .......... 1380
KF539852.1:c99164-96948    .......... .......... .......... .......... .......... .......... 1380
                                                   1,400                1,420                1,440
MK144329                   TTAGGAGCCG TGTGAGATGA AAGTCTCATG CACGGTTTTG AATGAGAGAA AGAAGTGAGG 1440
MH939982.1:c106394-104178  .......... .......... .......... .......... .......... .......... 1440
MH939981.1:c106661-104445  .......... .......... .......... .......... .......... .......... 1440
KF539850.1:c100173-97957   .......... .......... .......... .......... .......... .......... 1440
MG678915.1:c103141-100925  .......... .......... .......... .......... .......... .......... 1440
MG678876.1:c103509-101293  .......... .......... .......... .......... .......... .......... 1440
MG678856.1:c103368-101152  .......... .......... .......... .......... .......... .......... 1440
MG678843.1:c103267-101051  .......... .......... .......... .......... .......... .......... 1440
MG678835.1:c100962-98746   .......... .......... .......... .......... .......... .......... 1440
KF539853.1:c98719-96503    .......... .......... .......... .......... .......... .......... 1440
KF539852.1:c99164-96948    .......... .......... .......... .......... .......... .......... 1440
```

```
                                              1,460                    1,480                    1,500
                                                  |                        |                        |
               MK144329 AATCCTCTTT TCGACTCTGA CTCTCCCACT CCAGTCGTTG CTTTTCTTTC TGTTACTTCG 1500
MH939982.1:c106394-104178 .......... .......... .......... .......... .......... .......... 1500
MH939981.1:c106661-104445 .......... .......... .......... .......... .......... .......... 1500
 KF539850.1:c100173-97957 .......... .......... .......... .......... .......... .......... 1500
MG678915.1:c103141-100925 .......... .......... .......... .......... .......... .......... 1500
MG678876.1:c103509-101293 .......... .......... .......... .......... .......... .......... 1500
MG678856.1:c103368-101152 .......... .......... .......... .......... .......... .......... 1500
MG678843.1:c103267-101051 .......... .......... .......... .......... .......... .......... 1500
 MG678835.1:c100962-98746 .......... .......... .......... .......... .......... .......... 1500
   KF539853.1:c98719-96503 .......... .......... .......... .......... .......... .......... 1500
   KF539852.1:c99164-96948 .......... .......... .......... .......... .......... .......... 1500
                                              1,520                    1,540                    1,560
                                                  |                        |                        |
               MK144329 AAAGTAGCTG CTTCAGCTTT AGCCACTCGA ATTTTCGATA TTCCTTTTTA TTTCTCATCA 1560
MH939982.1:c106394-104178 .......... .......... .......... .......... .......... .......... 1560
MH939981.1:c106661-104445 .......... .......... .......... .......... .......... .......... 1560
 KF539850.1:c100173-97957 .......... .......... .......... .......... .......... .......... 1560
MG678915.1:c103141-100925 .......... .......... .......... .......... .......... .......... 1560
MG678876.1:c103509-101293 .......... .......... .......... .......... .......... .......... 1560
MG678856.1:c103368-101152 .......... .......... .......... .......... .......... .......... 1560
MG678843.1:c103267-101051 .......... .......... .......... .......... .......... .......... 1560
 MG678835.1:c100962-98746 .......... .......... .......... .......... .......... .......... 1560
   KF539853.1:c98719-96503 .......... .......... .......... .......... .......... .......... 1560
   KF539852.1:c99164-96948 .......... .......... .......... .......... .......... .......... 1560
                                              1,580                    1,600                    1,620
                                                  |                        |                        |
               MK144329 AACGAATGGC ATCTTCTTCT GGAAATCCTA GCTATTCTTA GCATGATATT GGGAAATCTC 1620
MH939982.1:c106394-104178 .......... .......... .......... .......... .......... .......... 1620
MH939981.1:c106661-104445 .......... .......... .......... .......... .......... .......... 1620
 KF539850.1:c100173-97957 .......... .......... .......... .......... .......... .......... 1620
MG678915.1:c103141-100925 .......... .......... .......... .......... .......... .......... 1620
MG678876.1:c103509-101293 .......... .......... .......... .......... .......... .......... 1620
MG678856.1:c103368-101152 .......... .......... .......... .......... .......... .......... 1620
MG678843.1:c103267-101051 .......... .......... .......... .......... .......... .......... 1620
 MG678835.1:c100962-98746 .......... .......... .......... .......... .......... .......... 1620
   KF539853.1:c98719-96503 .......... .......... .......... .......... .......... .......... 1620
   KF539852.1:c99164-96948 .......... .......... .......... .......... .......... .......... 1620
                                              1,640                    1,660                    1,680
                                                  |                        |                        |
               MK144329 ATTGCTATTA CTCAAACAAG CATGAAACGT ATGCTTGCAT ATTCGTCCAT AGGTCAAATC 1680
MH939982.1:c106394-104178 .......... .......... .......... .......... .......... .......... 1680
MH939981.1:c106661-104445 .......... .......... .......... .......... .......... .......... 1680
 KF539850.1:c100173-97957 .......... .......... .......... .......... .......... .......... 1680
MG678915.1:c103141-100925 .......... .......... .......... .......... .......... .......... 1680
MG678876.1:c103509-101293 .......... .......... .......... .......... .......... .......... 1680
MG678856.1:c103368-101152 .......... .......... .......... .......... .......... .......... 1680
MG678843.1:c103267-101051 .......... .......... .......... .......... .......... .......... 1680
 MG678835.1:c100962-98746 .......... .......... .......... .......... .......... .......... 1680
   KF539853.1:c98719-96503 .......... .......... .......... .......... .......... .......... 1680
   KF539852.1:c99164-96948 .......... .......... .......... .......... .......... .......... 1680
                                              1,700                    1,720                    1,740
                                                  |                        |                        |
               MK144329 GGATATGTAA TTATTGGAAT AATTGTTGGA GACTCAAATG ATGGATATGC AAGCATGATA 1740
MH939982.1:c106394-104178 .......... .......... .......... .......... .......... .......... 1740
MH939981.1:c106661-104445 .......... .......... .......... .......... .......... .......... 1740
 KF539850.1:c100173-97957 .......... .......... .......... .......... .......... .......... 1740
MG678915.1:c103141-100925 .......... .......... .......... .......... .......... .......... 1740
MG678876.1:c103509-101293 .......... .......... .......... .......... .......... .......... 1740
MG678856.1:c103368-101152 .......... .......... .......... .......... .......... .......... 1740
MG678843.1:c103267-101051 .......... .......... .......... .......... .......... .......... 1740
 MG678835.1:c100962-98746 .......... .......... .......... .......... .......... .......... 1740
   KF539853.1:c98719-96503 .......... .......... .......... .......... .......... .......... 1740
   KF539852.1:c99164-96948 .......... .......... .......... .......... .......... .......... 1740
                                              1,760                    1,780                    1,800
                                                  |                        |                        |
               MK144329 ACTTATATGC TGTTCTATAT CTCCATGAAT CTAGGAACTT TTGCTTGCAT TGTATTATTT 1800
MH939982.1:c106394-104178 .......... .......... .......... .......... .......... .......... 1800
MH939981.1:c106661-104445 .......... .......... .......... .......... .......... .......... 1800
 KF539850.1:c100173-97957 .......... .......... .......... .......... .......... .......... 1800
MG678915.1:c103141-100925 .......... .......... .......... .......... .......... .......... 1800
MG678876.1:c103509-101293 .......... .......... .......... .......... .......... .......... 1800
MG678856.1:c103368-101152 .......... .......... .......... .......... .......... .......... 1800
MG678843.1:c103267-101051 .......... .......... .......... .......... .......... .......... 1800
 MG678835.1:c100962-98746 .......... .......... .......... .......... .......... .......... 1800
   KF539853.1:c98719-96503 .......... .......... .......... .......... .......... .......... 1800
   KF539852.1:c99164-96948 .......... .......... .......... .......... .......... .......... 1800
```

```
                                        1,820                    1,840                    1,860
                                        |                        |                        |
MK144329                   GGTCTACGTA CCGGAACGGA TAACATTCGA GATTATGCAG GATTATACAC GAAAGATCCT 1860
MH939982.1:c106394-104178  .......... .......... .......... .......... .......... .......... 1860
MH939981.1:c106661-104445  .......... .......... .......... .......... .......... .......... 1860
KF539850.1:c100173-97957   .......... .......... .......... .......... .......... .......... 1860
MG678915.1:c103141-100925  .......... .......... .......... .......... .......... .......... 1860
MG678876.1:c103509-101293  .......... .......... .......... .......... .......... .......... 1860
MG678856.1:c103368-101152  .......... .......... .......... .......... .......... .......... 1860
MG678843.1:c103267-101051  .......... .......... .......... .......... .......... .......... 1860
MG678835.1:c100962-98746   .......... .......... .......... .......... .......... .......... 1860
KF539853.1:c98719-96503    .......... .......... .......... .......... .......... .......... 1860
KF539852.1:c99164-96948    .......... .......... .......... .......... .......... .......... 1860
                                        1,880                    1,900                    1,920
                                        |                        |                        |
MK144329                   TTTTTGGCTC TCTCTTTAGC CCTATGTCTC TTATCCCTAG GGGGTCTTCC CCCACTAGCA 1920
MH939982.1:c106394-104178  .......... .......... .......... .......... .......... .......... 1920
MH939981.1:c106661-104445  .......... .......... .......... .......... .......... .......... 1920
KF539850.1:c100173-97957   .......... .......... .......... .......... .......... .......... 1920
MG678915.1:c103141-100925  .......... .......... .......... .......... .......... .......... 1920
MG678876.1:c103509-101293  .......... .......... .......... .......... .......... .......... 1920
MG678856.1:c103368-101152  .......... .......... .......... .......... .......... .......... 1920
MG678843.1:c103267-101051  .......... .......... .......... .......... .......... .......... 1920
MG678835.1:c100962-98746   .......... .......... .......... .......... .......... .......... 1920
KF539853.1:c98719-96503    .......... .......... .......... .......... .......... .......... 1920
KF539852.1:c99164-96948    .......... .......... .......... .......... .......... .......... 1920
                                        1,940                    1,960                    1,980
                                        |                        |                        |
MK144329                   GGTTTTTTCG GAAAACTCTA TTTATTCTGG TGTGGATGGC AGGCAGGCCT ATATTTCTTG 1980
MH939982.1:c106394-104178  .......... .......... .......... .......... .......... .......... 1980
MH939981.1:c106661-104445  .......... .......... .......... .......... .......... .......... 1980
KF539850.1:c100173-97957   .......... .......... .......... .......... .......... .......... 1980
MG678915.1:c103141-100925  .......... .......... .......... .......... .......... .......... 1980
MG678876.1:c103509-101293  .......... .......... .......... .......... .......... .......... 1980
MG678856.1:c103368-101152  .......... .......... .......... .......... .......... .......... 1980
MG678843.1:c103267-101051  .......... .......... .......... .......... .......... .......... 1980
MG678835.1:c100962-98746   .......... .......... .......... .......... .......... .......... 1980
KF539853.1:c98719-96503    .......... .......... .......... .......... .......... .......... 1980
KF539852.1:c99164-96948    .......... .......... .......... .......... .......... .......... 1980
                                        2,000                    2,020                    2,040
                                        |                        |                        |
MK144329                   GTTTTAATAG GACTCCTTAC AAGCGTTGTT TCTATCTACT ATTATCTAAA AATAATCAAG 2040
MH939982.1:c106394-104178  .......... .......... .......... .......... .......... .......... 2040
MH939981.1:c106661-104445  .......... .......... .......... .......... .......... .......... 2040
KF539850.1:c100173-97957   .......... .......... .......... .......... .......... .......... 2040
MG678915.1:c103141-100925  .......... .......... .......... .......... .......... .......... 2040
MG678876.1:c103509-101293  .......... .......... .......... .......... .......... .......... 2040
MG678856.1:c103368-101152  .......... .......... .......... .......... .......... .......... 2040
MG678843.1:c103267-101051  .......... .......... .......... .......... .......... .......... 2040
MG678835.1:c100962-98746   .......... .......... .......... .......... .......... .......... 2040
KF539853.1:c98719-96503    .......... .......... .......... .......... .......... .......... 2040
KF539852.1:c99164-96948    .......... .......... .......... .......... .......... .......... 2040
                                        2,060                    2,080                    2,100
                                        |                        |                        |
MK144329                   TTATTAATGA CTGGACGAAA CCAAGAAATA ACCCCTCACG TGCGAAATTA TAGAAGATCT 2100
MH939982.1:c106394-104178  .......... .......... .......... .......... .......... .......... 2100
MH939981.1:c106661-104445  .......... .......... .......... .......... .......... .......... 2100
KF539850.1:c100173-97957   .......... .......... .......... .......... .......... .......... 2100
MG678915.1:c103141-100925  .......... .......... .......... .......... .......... .......... 2100
MG678876.1:c103509-101293  .......... .......... .......... .......... .......... .......... 2100
MG678856.1:c103368-101152  .......... .......... .......... .......... .......... .......... 2100
MG678843.1:c103267-101051  .......... .......... .......... .......... .......... .......... 2100
MG678835.1:c100962-98746   .......... .......... .......... .......... .......... .......... 2100
KF539853.1:c98719-96503    .......... .......... .......... .......... .......... .......... 2100
KF539852.1:c99164-96948    .......... .......... .......... .......... .......... .......... 2100
                                        2,120                    2,140                    2,160
                                        |                        |                        |
MK144329                   CCTTTAAGAT CAAACAATTC CATCGAATTG AGTATGATTG TATGTGTAAT AGCATCTACT 2160
MH939982.1:c106394-104178  .......... .......... .......... .......... .......... .......... 2160
MH939981.1:c106661-104445  .......... .......... .......... .......... .......... .......... 2160
KF539850.1:c100173-97957   .......... .......... .......... .......... .......... .......... 2160
MG678915.1:c103141-100925  .......... .......... .......... .......... .......... .......... 2160
MG678876.1:c103509-101293  .......... .......... .......... .......... .......... .......... 2160
MG678856.1:c103368-101152  .......... .......... .......... .......... .......... .......... 2160
MG678843.1:c103267-101051  .......... .......... .......... .......... .......... .......... 2160
MG678835.1:c100962-98746   .......... .......... .......... .......... .......... .......... 2160
KF539853.1:c98719-96503    .......... .......... .......... .......... .......... .......... 2160
KF539852.1:c99164-96948    .......... .......... .......... .......... .......... .......... 2160
```

		2,180		2,200			
MK144329	ATACCAGGAA	TATCAATGAA	CCCGATTATT	GCAATTGCTC	AGGATACACT	TTTTTAG	2217
MH939982.1:c106394-104178	....	....	....	....	....	....	2217
MH939981.1:c106661-104445	....	....	....	....	....	....	2217
KF539850.1:c100173-97957	....	....	....	....	....	....	2217
MG678915.1:c103141-100925	....	....	....	....	....	....	2217
MG678876.1:c103509-101293	....	....	....	....	....	....	2217
MG678856.1:c103368-101152	....	....	....	....	....	....	2217
MG678843.1:c103267-101051	....	....	....	....	....	....	2217
MG678835.1:c100962-98746	....	....	....	....	....	....	2217
KF539853.1:c98719-96503	....	....	....	....	....	....	2217
KF539852.1:c99164-96948	....	....	....	....	....	....	2217
