# Supplementary material for: Light/heat effects on RNA editing in chloroplast NADH-plastoquinone oxidoreductase subunit 2 (ndhB) gene of Calotropis (Calotropis procera)
Source: J Genet Eng Biotechnol. 2020 Sep 11;18:49. doi: 10.1186/s43141-020-00064-4 (PMC7486354; doi:10.1186/s43141-020-00064-4)
Supplement: Supplementary file 3 — Additional file 3: Fig S2. A comparison between ndhB sequences of the genomic DNA and 3 times cDNA revealed RNA editing sites. [file 43141_2020_64_MOESM3_ESM.docx]

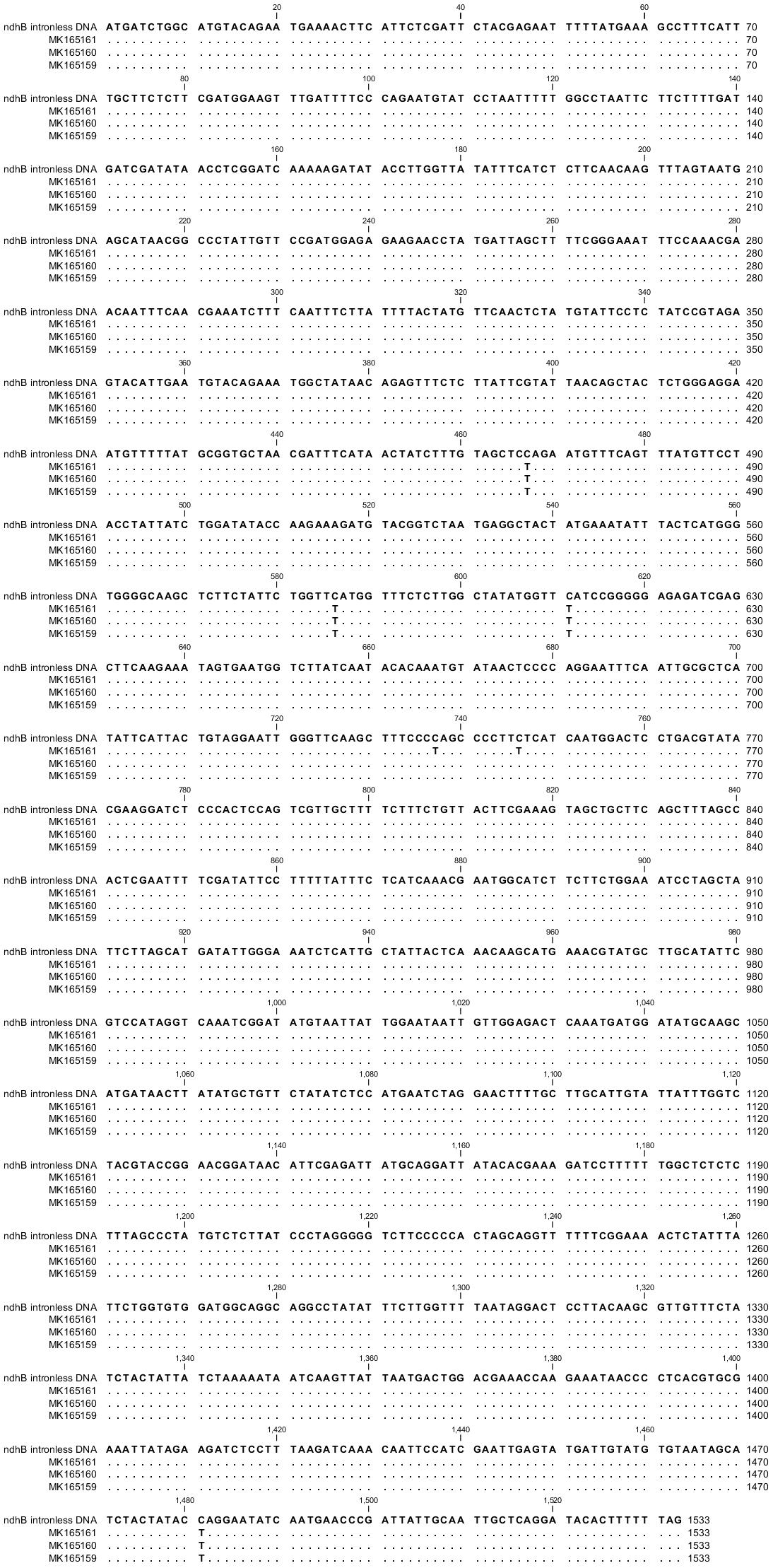
Fig S2. A comparison between *ndhB* sequences of the genomic DNA and 3 times cDNA revealed RNA editing sites
